# Challenges and success stories of the implementation of infection control and antimicrobial stewardship strategies: proceedings of the 5th Global Ministerial Summit on Patient Safety, 2023

**DOI:** 10.1186/s13756-023-01344-7

**Published:** 2024-02-08

**Authors:** Andrea C. Büchler, Murielle Haddad Galas, Niccolò Buetti, Emine Alp, Anucha Apisarnthanarak, Gerald Dziekan, Valeria Fabre, Simon Gottwalt, Kazuaki Jindai, Babacar Ndoye, Hilda Márquez Villareal, Fernando Otaiza, Didier Pittet, Natalie Schellack, Céline Gardiol, Stephan Harbarth

**Affiliations:** 1 Infection Control Program, Faculty of Medicine, Geneva University Hospitals, WHO Collaborating Center, Rue Gabrielle-Perret-Gentil 4, Genève, 1205 Switzerland; 2 IAME UMR 1137, INSERM, Université Paris-Cité, Paris, France; 3 Department of Infectious Diseases and Clinical Microbiology, Faculty of Medicine, Ankara Yıldırım Beyazıt University, Ankara, Türkiye; 4 Division of Infectious Diseases, Faculty of Medicine, Thammasat University Hospital, Pathum Thani, Thailand; 5 Communicable Disease Division, Federal Office of Public Health FOPH, Bern, Switzerland; 6 Department of Medicine, Division of Infectious Diseases, Johns Hopkins University School of Medicine, Baltimore, USA; 7 Department of Virology, Tohoku University Graduate School of Medicine, Sendai, Japan; 8 Department of Healthcare Epidemiology, School of Public Health, Kyoto University, Kyoto, Japan; 9 Infection Control and Patient Safety, WHO Afro Consultant, Brazzaville, Congo Republic; 10 Department of Public Health. University Center of Health Sciences, University of Guadalajara, Guadalajara, Mexico; 11 Department of Quality of Healthcare and Patient Safety, National Infection Control Program, Ministry of Health of Chile, Santiago, Chile; 12 Departement of Pharmacology, Faculty of Health Sciences, University of Pretoria, Pretoria, South Africa

**Keywords:** Infection control, Antibiotic stewardship, Antimicrobial resistance, Health plan implementation, Global ministerial summit on patient safety

## Abstract

The 5th edition of the Global Ministerial Summit on Patient Safety was held in Montreux, Switzerland, in February 2023, delayed by three years due to the COVID-19 pandemic. The overarching theme of the summit was “*Less Harm, Better Care – from Resolution to Implementation*”, focusing on the challenges of implementation of infection prevention and control (IPC) strategies as well as antimicrobial stewardship programs (ASP) around the world. IPC strategies and ASP are of increasing importance due to the substantial burden of healthcare-associated infections and antimicrobial resistance threatening patient safety. Here, we summarize countries’ and regional experiences and activities related to the implementation of IPC strategies and ASP shared at the meeting. Full implementation of effective programs remains a major challenge in all settings due to limited support by political and healthcare leaders, and human and financial constraints. In addition, the COVID-19 pandemic challenged already well-established programs. By enforcing sustained implementation by dedicated, cross-disciplinary healthcare personnel with a broad skill set, a reduction in healthcare-associated infections and multidrug-resistant pathogens can be achieved, leading ultimately to improved patient safety.

## Introduction

In 2016, the first edition of the Global Ministerial Summit on Patient Safety was held in London. Over the years, these summits have been raising awareness for this crucial public health issue by bringing together political leaders with experts of the field. The 5th edition of the Global Ministerial Summit on Patient Safety was held in Montreux, Switzerland, in February 2023. The overarching theme of the summit was “*Less Harm, Better Care – from Resolution to Implementation*”. The event covered a wide spectrum of patient safety related topics including challenges of implementation of infection prevention and control (IPC) strategies as well as antimicrobial stewardship programs (ASP) around the world. Over 500 international participants with various backgrounds such as medical professionals, civil servants, patient representatives and academia shared their insights on sustainable implementation of patient safety measures with official high-level delegations from about 80 countries from all regions around the world.

IPC strategies and ASP are of high importance due to the substantial burden of healthcare-associated infections (HAI) and antimicrobial resistance (AMR) threatening patient safety. HAI are the most frequent adverse event in healthcare, affecting 5–15% of patients in acute care hospitals, leading to high morbidity and mortality with incalculable economic costs [1–3]. AMR is an additional major menace, listed as one of the ten most urgent health threats in 2019 by the World Health Organization (WHO) [4]. Recently, it was estimated that 1.3 million deaths attributable to AMR occurred in 2019 [5]. The implementation of effective prevention strategies for AMR is ongoing, focusing mostly on improved hand hygiene, ASP, IPC strategies, and environmental hygiene, but also improving development of and access to rapid diagnostics and prescriptions as well as mass media campaigns.

This paper summarizes the main proceedings of eight countries and regions from different parts of the world, who were invited to this Global Ministerial Summit on Patient Safety to report their experiences on the implementation of IPC strategies and ASP with the overarching aim to reduce the burden of HAI and AMR in various healthcare settings.

## Implementation of IPC strategies and ASP: challenges and successes

### Implementing IPC core components: experiences from four countries

The WHO global strategy on IPC is based on two main pillars: political action, supported by the World Health Assembly resolution “Global Strategy on IPC” of 2022 [6], and healthcare facility-centered action. The latter is based on the eight core components recommended by the WHO [7] (Table 1). However, uncertainty exists about real-life implementation of the WHO resolution as efforts might be variable. Four countries provided an insight in their practices at the 5th Global Ministerial Summit on Patient Safety 2023. The overview of the country experiences is presented in Table 2.

**Table 1:** Summary of the eight core components for infection prevention and control programs by the World Health Organization (WHO) with focus on healthcare-facility level recommendations. Table adapted from https://www.who.int/teams/integrated-health-services/infection-prevention-control/core-components. IPC: infection prevention and control; AMR: antimicrobial resistance; HAI: healthcare-associated infections

	Core component by WHO	Recommendation and good practice statement
1	IPC programs	IPC program with a dedicated, trained team should be in place in each acute health care facility for the purpose of preventing HAI and combating AMR through good IPC practices.
2	IPC guidelines	Evidence-based guidelines should be developed and implemented for the purpose of reducing HAI and AMR. The education and training of relevant health care workers on the guideline recommendations and the monitoring of adherence with guideline recommendations should be undertaken to achieve successful implementation
3	IPC education and training	IPC education should be in place for all health care workers by utilizing team- and task-based strategies that are participatory and include bedside and simulation training to reduce the risk of HAI and AMR.
4	Surveillance	Facility-based HAI surveillance should be performed to guide IPC interventions and detect outbreaks, including AMR surveillance with timely feedback of results to health care workers and stakeholders and through national networks.
5	Multimodal strategies	IPC activities using multimodal strategies should be implemented to improve practices and reduce HAI and AMR.
6	Monitoring /audit of IPC practices and feedback	Regular monitoring/audit and timely feedback of health care practices according to IPC standards should be performed to prevent and control HAI and AMR at the health care facility level. Feedback should be provided to all audited persons and relevant staff.
7	Workload, staffing and bed occupancy (acute health care facility only)	The following elements should be adhered to in order to reduce the risk of HAI and the spread of AMR: (1) Bed occupancy should not exceed the standard capacity of the facility. (2) Health care worker staffing levels should be adequately assigned according to patient workload.
8	Built environment, materials, and equipment for IPC at the facility level (acute health care facility only)	Patient care activities should be undertaken in a clean and/or hygienic environment that facilitates practices related to the prevention and control of HAI, as well as AMR, including all elements around the WASH infrastructure and services and the availability of appropriate IPC materials and equipment. Materials and equipment to perform appropriate hand hygiene should be readily available at the point of care.

**Table 2:** Implementing the core components for infection prevention and control: Four country experiences from all over the world with focus on successes and challenges. USD: United States Dollar. IPC: infection prevention and control, HAI: healthcare-associated infections, COVID-19 Coronavirus Disease 2019

Country	Healthcare costs per capita in USD (year) [8]	Global Health Security Index 2021 [50]	Established infection prevention and control program	Success	Challenges
Senegal	77 (2020)	Overall: 32.8 Prevent: 11.0 Detect: 28.3 Respond: 41.3 Health:14.6 Norms: 54.0 Risk: 47.8	National IPC program introduced in 2004	Overall performance of 52% of the minimum requirements. Well established IPC program at all levels of the healthcare system with a national budget	Lack of implementation and operational problems after more than a decade of active IPC program
Mexico	539 (2020)	Overall: 57.0 Prevent: 41.9 Detect: 54.3 Respond: 64.8 Health: 54.7 Norms: 68.1 Risk: 57.9	15 IPC initiatives: - 6 regulatory frameworks - 9 manuals or guidelines	Sentinel Surveillance System for HAI since 1997. Local and individual achievements by institutions or universities.	Development and implementation of collaborative strategies and overall reduction of HAI
Chile	1,479 (2021)	Overall: 56.2 Prevent: 47.2 Detect: 58.1 Respond: 59.5 Health: 52.9 Norms: 53.1 Risk: 66.2	National IPC program introduced in 1983 (mandatory since 1986)	Well established IPC program since decades.	COVID-19 pandemic with increase in HAI outbreaks and incidence of multidrug-resistant microorganisms while human resources were drastically reduced
Türkiye	395 (2020)	Overall: 50.0 Prevent: 51.1 Detect: 41.4 Respond: 36.6 Health: 53.9 Norms: 59.7 Risk: 57.2	National IPC program introduced in 2005 National action plan for prevention and control of antimicrobial resistance and sepsis introduced in 2019	Well established surveillance system for healthcare-associated infections and multidrug-resistant microorganisms.	Implementation of multimodal strategies, improving workload and staffing as well as bed occupancy

### Senegal

Senegal’s healthcare system is predominantly public. The healthcare costs were 77 USD per capita in 2020 [8]. Since 2004, a national IPC program has been progressively implemented. The main achievements of the program have been to train healthcare workers in a specialized training facility, the establishment of IPC committees in all hospitals, the completion of three national HAI prevalence surveys, a dedicated program to improve hand hygiene and basic hygiene measures, as well as the regulation of the healthcare waste management. Unfortunately, the activities were not sustainable after the withdrawal of funding provided by external partners due to lack of human, material, and operational resources. In 2017, after 13 years of an active IPC program, Senegal achieved an overall performance score of 15% in the IPCAT2 WHO tool [9], during an international IPC training workshop, showing lack of implementation. However, in the following five years, the overall performance increased to 52% of the minimum requirements with an increase in IPC program and guideline implementation, education and training, surveillance, and multimodal strategies. It should be noted that during this period, the majority of activities implemented were financed and directed by partners, instead of following a well-structured national action plan with well-defined objectives, strategies and indicators. This last assessment was carried out during a national workshop held in 2022, which also helped to develop a new IPC strategic plan for the period 2023–2027. However, these results need to be interpreted with caution, as it compares the full IPC requirements to the minimum requirements, and the latter was assessed by a self-assessment approach possibly overestimating the performance.

### Mexico

In Mexico, the healthcare system is integrated by different public and private institutions and different amendments are currently being made to the model of care. The healthcare costs were 539 USD per capita in 2020 [8]. There is no overarching national IPC strategy, but 15 IPC initiatives are currently promoted including 6 regulatory frameworks and 9 manuals or guidelines [10]. Examples for these IPC initiatives are the national standard for epidemiological surveillance and a manual of standardized procedures for hospital surveillance. The biggest challenges in patient safety experienced in Mexico are the development and implementation of collaborative strategies and the overall reduction of HAI, whose attributable case-fatality rate is estimated to be around 5%. Due to the complex structure of the healthcare system, there are multiple initiatives to improve IPC, but continuous and unified education and training as well as implementation remains a challenge. This challenge is mainly addressed by local efforts by institutions or universities, which are often not shared across the country or even disseminated in scientific journals. Monitoring of IPC tools and effects are variable and inconsistent, and actions are not always sustainable.

### Chile

Chile’s healthcare system is mostly based on public institutions, covering roughly two thirds of the yearly hospitalizations. The healthcare costs were 1,479 USD per capita in 2021 [8]. Already in 1983, Chile implemented a comprehensive national IPC program involving all public and private sectors. The IPC program became mandatory for all healthcare facilities in 1986. During the COVID-19 pandemic, Chile’s healthcare system was heavily challenged by an increased number of outbreaks of HAI including outbreaks caused by multidrug-resistant bacteria, leading to a higher AMR incidence [11, 12]. Managing these outbreaks was challenging due to an unexpected high number of patients and healthcare workers affected by COVID-19 itself leading to staff shortages. Despite the great progress in reducing the incidence of HAI during the last 25 years [13–15], almost all types of HAI increased during the pandemic. Despite these challenges, Chile managed to train an eight-fold higher number of healthcare workers in IPC during 2020 compared to previous years. The training focused on standard precaution measures, the use of personal protective equipment and hand hygiene, but also covered more specific topics such as outbreak management and prevention of ventilator-associated pneumonia. In online and short face-to-face sessions, healthcare workers were educated and tools for the assessment of IPC needs were introduced. Feedback was provided to the healthcare providers every 4–6 months, also offering practical solutions.

### Türkiye

In Türkiye, the healthcare system is covered by public funding by roughly 75% based on the number of beds available [16]. In 2020, healthcare costs were 395 USD per capita [8]. The most important patient safety challenge in Türkiye during the past years was the high incidence of HAI, illustrated by the rates of ventilator-associated events, catheter-associated urinary tract infections, and central line-associated bloodstream infections of 17.7, 5.0, and 5.7 per 1000 device-days, respectively, in 2008, which already decreased to 4.9, 1.6, and 2.8 per 1000 device-days, respectively, in 2017 [17]. In 2005, a structured IPC program has been put in place on a national scale, covering detailed IPC strategies, training, web-based surveillance, and promotion of hand hygiene [17]. Since then, the rate of HAI has been reduced significantly in hospitals of all sizes [17]. In addition to this success, the Ministry of Health focused on the implementation of more core components, such as a general system and culture change, training and education, as well as monitoring and feedback. This resulted in an advanced IPC level in 73.5% of all healthcare facilities as shown by the IPCAF results in 2019 [18]. As remaining issues, multimodal strategies, workload, staffing, and bed occupancy were identified. In addition, AMR remains an endemic problem in Türkiye, with carbapenem-resistant Gram-negatives in 10–90% of all infecting isolates depending on the respective microorganisms and settings. To combat this, a national action plan for prevention and control of AMR and sepsis has been launched in 2019.

### Challenges in implementing ASP, perspectives from three countries and one region

The main pillar of combatting AMR remains ASP. The main objective of ASP is to improve clinical outcomes and patient safety by ensuring the right antimicrobial is given to the right patient at the right time, for the right duration, in a cost-effective manner [19]. The core elements of an ASP are: leadership commitment, accountability, pharmacy expertise, action to improve antibiotic use, monitoring and reporting of antibiotic use and AMR rates, and education [20]. An overview of the selected country and regional experiences is presented in Table 3.

**Table 3:** Challenges in implementing an antibiotic stewardship program in four countries or regions. AMR: antimicrobial resistance, ASP: antibiotic stewardship program, TrACSS: Tracking AMR Country Self-Assessment Survey, IPC: infection prevention and control, EML: essential medicine list, NA: not available. TrACSS Country Report grading: A: none, B: limited, C: developed, D: demonstrated, E: sustained

Country/Region	AMR burden	TrACSS 2022 Country reports [51]	Established antimicrobial stewardship program	Success	Challenges
Thailand	Estimated to cause 88,000 infections, 3.24 additional hospitalizations, and 38,000 deaths in 2010 (21)	Training and Education on AMR in human healthcare sector: C Monitoring antimicrobial consumption in human health C National surveillance system for AMR in human health: D IPC in human health: D Adoption of AWaRe classification into national EML: D [52]	National AMR Surveillance Centre founded in 1998 Additional antibiotics smart use program added in 2007 Antibiotic awareness day introduced 2013	80% of hospitals have an established ASP	Evaluation of effectiveness of ASP is ongoing
Japan	Disability-adjusted life years due to bloodstream infections caused by nine major antimicrobial-resistant bacteria: 195.2/100,000 population (2021) [53]	Training and Education on AMR in human healthcare sector: E Monitoring antimicrobial consumption in human health E National surveillance system for AMR in human health: E IPC in human health: E Adoption of AWaRe classification into national EML: NA [54]	Action plan on antimicrobial resistance introduced in 2016 Updated national action plan on antimicrobial resistance in 2023	Immediate effect seen on use of antimicrobials	No sustainable effect, despite ongoing downward trend in use of antimicrobials
South Africa	According to the Global Burden of Disease study, sub-Saharan Africa had the highest mortality (23.7 deaths per 100,000) attributable to AMR (5).	Training and Education on AMR in human healthcare sector: C Monitoring antimicrobial consumption in human health B National surveillance system for AMR in human health: D IPC in human health: B Adoption of AWaRe classification into national EML: B [55]	Antimicrobial resistance National Strategy Framework introduced in 2014 (2014–2024) Guidelines on Implementation of the Antimicrobial Strategy in South Africa: One Health Approach & Governance (February 2017) South African Antimicrobial Resistance National Strategy Framework; a one Heath approach (2018–2024): Guidelines for the Prevention and Containment of AMR in South African Hospitals: One Health Approach and Governance 2018	Ministerial Advisory Board for Antimicrobial Resistance has been appointed by the Minister of Health, in 2015. A nationwide surveillance of antimicrobial use by a web-based application.	South Africa’s national action plan against antibiotic resistant bacterial infections remains unfunded. The prevalence of infections caused by difficult-to-treat resistant Gram-negative bacteria (DTR-GNB) is rapidly increasing.
Latin America	Estimated to cause 338,000 deaths associated with and 84,300 deaths attributable to AMR in Latin America and the Caribbean (5).	NA	In 22 out of 33 countries in Latin America, 50% have not implemented an ASP yet, whereas in the other half, an ASP is currently being implemented.	Cost-effectiveness of ASP in Latin America are proven	Limited support by federal governments and local healthcare facility administrators to establish resources for ASP

### Thailand

AMR was estimated to cause 88,000 infections resulting in 3.24 million additional hospitalization days and 38,000 deaths in Thailand in 2010 [21]. The first IPC program was launched in 1971, augmented by a national AMR Surveillance Centre in 1998. In 2007, the *antibiotics smart use* program was started with the aim to reduce unnecessary antibiotic use for common self-limiting diseases, such as upper respiratory tract infections. To raise additional public awareness, an antibiotic awareness day has been introduced in 2013 [22]. The Thai strategy for tackling AMR includes six strategies covering governance mechanisms to implement and sustain AMR actions, AMR surveillance using a One Health approach, IPC and ASP, regulation of antimicrobial distribution, public awareness, and antimicrobial use in agriculture and animals [23]. As a result of these efforts, more than 80% of Thai hospitals have an established, multi-disciplinary ASP [24]. Diagnostic stewardship is more frequently performed in hospitals with a broader expertise in antimicrobial stewardship. The evaluation of the effectiveness of these programs is still ongoing.

### Japan

As of 2016, oral antimicrobials accounted for > 90% of antimicrobials prescribed over the past decade in Japan; and the younger generation, particularly those aged ≤ 15 years, were prescribed more antimicrobials than adults [25]. As a reaction to it, a national AMR action plan was introduced in 2016 focusing on clinical guidance for acute respiratory tract infections and diarrhea, financial incentives for pediatricians and family physicians, and communication of risks to the public. By focusing on the first two, an immediate reduction in the outpatient antimicrobial prescription rate was observed [26]. Nevertheless, the effect was not sustainable. Even though a steady downward trend could be shown in the overall use of antimicrobials [25], the targeted reduction of 33% was not achieved until 2020. It remains unknown to what extent this change was based on the reduction of inappropriate prescribing. To better understand the appropriateness of antimicrobial use in Japan, more detailed quantitative and qualitative investigations and surveillance systems are needed as a next step. The updated national action plan was published in 2023. The government of Japan continues to tackle the AMR issues through synergetic collaboration with multiple sectors, namely by strengthening and implementing integrated One Health surveillance on humans, animals, food, and the environment. Furthermore, in addition to human health and the food production sector, the updated national action plan also emphasizes ASP in veterinary medicine [27].

### South Africa

South Africa approved a national AMR Strategy Framework (2014–2024) and consecutively implemented the following key components: definition of measurable goals to track progress and evaluate the effectiveness, a pharmacist-driven prospective audit and feedback strategy, prescription audits and usage, antimicrobial formulary management, IPC programs, and clinical workforce education [28]. The public healthcare that serves 86% of the population in South Africa still mostly uses a paper-based system. A situational analysis was conducted on antimicrobial utilization and policies. Public sector data were obtained from contracting data arising from tenders from wholesalers where the National Department of Health solicits bids from suppliers. Antimicrobial use increased from 2013 to 2018, especially oral broad-spectrum penicillins, oral and intravenous cephalosporins, and certain reserve antibiotics, such as daptomycin, linezolid, and tigecycline. The latter is partly explainable be the emergence of multidrug-resistant tuberculosis requesting therapy with these antibiotics. Unfortunately, a sub-optimal compliance to the strategy by the public healthcare sector has been observed [29]. As a result, the creation of interdisciplinary teams including microbiologists, infection preventionists, nurses, pharmacists, and infectious diseases specialists were supported. Adapted to the local characteristics, a web-based application to collect data on antimicrobial utilization has been developed, improving the overall surveillance of antibiotic consumption [30]. Future targets to improve antimicrobial usage were also identified: use of watch antibiotics, surgical prophylaxis, and extended antimicrobial prophylaxis amongst others.

### Latin America

Data from cross-sectional studies showed that ASPs in Latin American healthcare facilities currently face various problems, including limited leadership support, and dedicated, multi-disciplinary staff for an effective ASP including infectious disease trained pharmacists [31–33]. Furthermore, cultural and behavioral determinants pose a risk to sustainable ASP implementation as power distance and hierarchical relationships limit integration of pharmacists and non-physician roles in AS activities. While ASP are proven to decrease antimicrobial consumption on a facility level [34], data on national levels is missing for most Latin American countries. Even though studies support the (cost-) effectiveness of ASPs in Latin American countries [35, 36], further actions from the federal governments are needed to create the necessary resources to establish and implement effective ASPs, and for hospital leaders to make initial investments to set effective ASPs in motion. Several targets for improving antibiotic use in acute care hospitals have been identified including treatment of HAIs in the ICU, empiric treatments, adherence to treatment guidelines, and use of broad-spectrum antibiotics [37]. Given the increase in carbapenem resistance among Gram-negatives in the region in recent decades there is an urgent need to strengthen both IPC and ASP in hospitals in the region.

## Discussion

The implementation of the WHO IPC core components and ASP has been recognized as an essential step to battle the emerging burden of HAI and AMR. Experts from different countries and regions with different income levels gave their insights into their successes and problems in the implementation of national IPC programs. Even though their conditions differ significantly, from a well-established IPC program since decades in Chile to a bundle of different IPC initiatives in Mexico, most of them face problems in the continued implementation process. Starting with the sustainability of the programs and the financing in Senegal, other reports included the lack of coordination and issues of different initiatives in Mexico, and the COVID-19 pandemic challenging existing IPC structures due to increased workload while being understaffed at the same time in Chile. ASP as an integral part of any program to combat AMR were introduced in the last 15 years in several presenting countries. However, the effect of these programs is difficult to measure and interpret, as AMR is known to be influenced not only by human consumption of antibiotics, but also antibiotic use in livestock and environmental factors, such as wastewater treatment [38]. This is illustrated by the Japanese experience, where an immediate effect on human antibiotic consumption could be shown after the introduction of interventions, unfortunately without a sustainable effect. In South Africa, despite the extensive surveillance of antimicrobial use, an increase of antibiotic consumption was observed during the following five years. The reason for this remains unknown but is demanding further actions. In some regions of the world, essential parameters for estimating the effect of ASP on AMR are not routinely assessed, such as antimicrobial consumption, which makes it even more difficult to evaluate the effect of individual measures in these specific settings.

In the last decade, guidelines have been published by the WHO [39], the Centers for Disease Control and Prevention (CDC) [40], and the European Centre for Disease Prevention and control (ECDC) [41] on the requirements of effective ASP and IPC programs. However, the implementation of these guidelines remains a major challenge as the resources available depend on the individual setting. The basis for the successful implementation of IPC programs and ASP is the support of political and healthcare leaders through legislation and processes that facilitate creation of the necessary resources for these programs. Coordination of implementation within a country, but also within a healthcare institution, are key to efficiently use the available human and financial resources. Dedicated healthcare personnel with different skill sets are essential for effective IPC programs and ASP. While the introduction of automated or non-automated surveillance tools seems feasible in most settings, the implementation of more time-consuming activities, such as education and training, behavior change or multimodal strategies, remain challenging. However, these activities are key to spread knowledge about IPC and ASP among healthcare workers and get them engaged and motivated. There is a clear need to expand the clinical pharmacy workforce in the inpatient setting as pharmacists have unique skill sets that are complementary to the expertise physicians have for optimal management of infectious diseases.

In contrast to hospital-based IPC programs, ASP face broader issues. As 80–90% of the antibiotic consumption takes place in the outpatient setting [42], there is a large, unmet need for promoting ASP in the ambulatory sector. Broad approaches for implementation are known in this setting, ranging from education of individual patients and general practitioners to electronic decision support tools to facilitate appropriate treatment decisions, and general education of the public [43]. As the targeted population is very broad, low participation and general time pressure are limiting factors of these approaches. Promising interventions are the introduction of small quality circles among primary care physicians, and the strengthening of the patient-physician relationship by point-of-care-testing and improved communication skills [44, 45].

Implementation science is still a young and developing field. Nevertheless, recent studies have shown that with the help of implementation specialists, evidence-based measures can be successfully introduced with increased sustainability [46, 47]. However, implementation is not a “one size fits all” model, as specific needs and expectations of involved healthcare personnel as well as setting specific characteristics need to be taken into account. As an example, the successful implementation of an IPC bundle to reduce central venous catheter-blood stream infections in Michigan, United States, could not be reproduced in another study conducted in England due to differences in the implementation process [48, 49].

In conclusion, the implementation of effective IPC and ASP guidelines remains a major challenge at all levels. Together, we can all work to reduce the burden of HAI and AMR. By enforcing sustained implementation by dedicated healthcare personnel with a broad skill set, a reduction in HAIs and multidrug-resistant pathogens can be achieved and, as a result, ultimately improve patient safety.

## Data Availability

Not applicable.

## Abbreviations

AMR: antimicrobial resistance
ASP: antibiotic stewardship program
CDC: Centers for Disease Prevention and Control
ECDC: European Centre for Disease Prevention and Control
EML: essential medicine list
HAI: healthcare-associated infections
IPC: infection prevention and control
TrACSS: Tracking AMR Country Self-Assessment Survey
WHO: World Health Organization
